# An Adaptable Metric Shapes Perceptual Space

**DOI:** 10.1016/j.cub.2016.05.047

**Published:** 2016-07-25

**Authors:** Rumi Hisakata, Shin’ya Nishida, Alan Johnston

**Affiliations:** 1 Department of Psychology, School of Human Sciences, Senshu University, 5G8, 2-1-1, Higashimita, Tama-ku, Kawasaki-shi, Kanagawa 214-8580, Japan; 2 NTT Communication Science Laboratories, Nippon Telegraph and Telephone Corporation, 3-1, Morinosato-Wakamiya, Atsugi-shi, Kanagawa 243-0198, Japan; 3 School of Psychology, University Park, The University of Nottingham, Nottingham NG7 2RD, UK

## Abstract

How do we derive a sense of the separation of points in the world within a space-variant visual system? Visual directions are thought to be coded directly by a process referred to as local sign, in which a neuron acts as a labeled line for the perceived direction associated with its activation [1, 2]. The separations of visual directions, however, are not given, nor are they directly related to the separations of signals on the receptive surface or in the brain, which are modified by retinal and cortical magnification, respectively [3]. To represent the separation of directions veridically, the corresponding neural signals need to be scaled in some way. We considered this scaling process may be influenced by adaptation. Here, we describe a novel adaptation paradigm, which can alter both apparent spatial separation and size. We measured the perceived separation of two dots and the size of geometric figures after adaptation to random dot patterns. We show that adapting to high-density texture not only increases the apparent sparseness (average element separation) of a lower-density pattern, as expected [4], but paradoxically, it reduces the apparent separation of dot pairs and induces apparent shrinkage of geometric form. This demonstrates for the first time a contrary linkage between perceived density and perceived extent. Separation and size appear to be expressed relative to a variable spatial metric whose properties, while not directly observable, are revealed by reductions in both apparent size and texture density.

## Highlights

• Adapting to high-density dot textures reduces the apparent separation of dot pairs
• It also induces an apparent shrinkage of geometric form
• Paradoxically, the same adaptor makes a dot texture appear sparser
• The compression effect cannot be ascribed to a window-based size aftereffect

## Graphical Abstract

**Figure:**
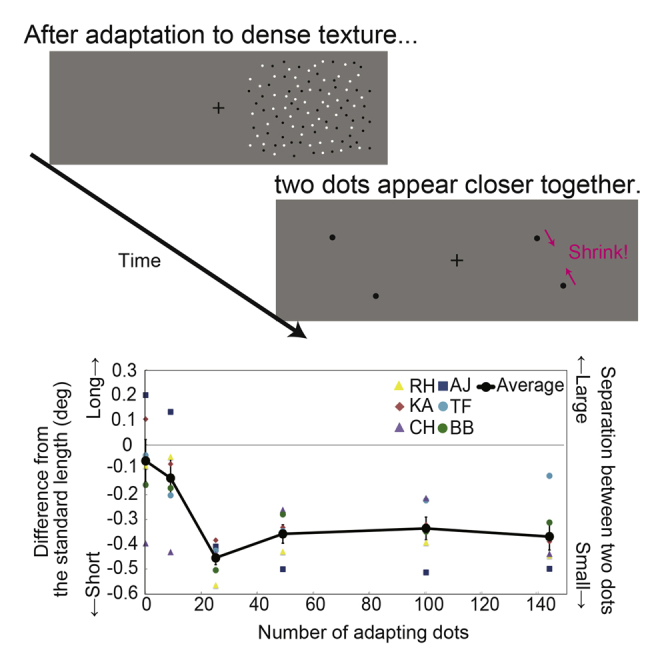

## Teaser

Hisakata et al. present a novel visual aftereffect that reveals the operation of a scaling mechanism within spatial vision. They show adaptation to dense texture reduces the apparent separation of subsequently presented dots pairs. This effect demonstrates that the visual system has an adaptable metric that controls our perception of visual space.

## Results and Discussion

**Figure 1:**
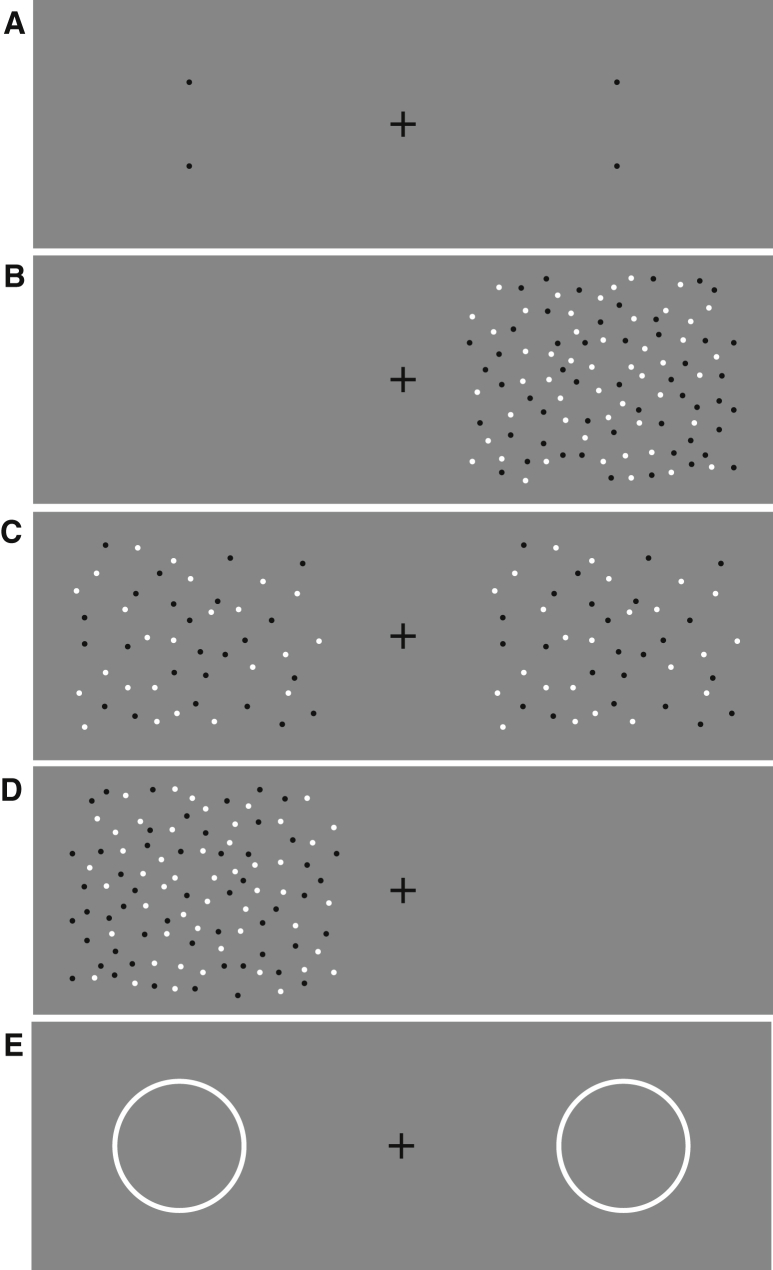
Demonstration of the Aftereffect (A–E) The adapting textures are shown in (B) and (D). After looking at the fixation cross in the adapting image for around 30 s, the perceived interval between the two test dots is reduced in the area adapted to the dense texture as compared to other side (A). Also the perceived size of a circle is reduced in the area adapted to the dense texture (E). However, perceived density decreases in the area adapted to the dense texture (C). The aftereffect occurs with a static adapting image, although it is larger when adapting to a dynamic random dot texture.

Figure 1 (see also Movie S1) provides a demonstration that adaptation to texture induces a change in the perceived distance between two dots (Figure 1A). After fixating the center cross in Figure 1B or Figure 1D for around 1 min, the interval between two dots (Figure 1A) on the side adapted to the higher dot density appears to be smaller than the comparison dots on the unadapted side. This is surprising, as it is well known that after adapting to a dense texture, an equal or less dense test texture presented in the adapted region appears more sparse [4, 5]. Apparent numerosity can also be affected [6, 7, 8].

The visual system might encode separation by selecting between fixed bi-local dot detectors acting as line elements for particular separations [9]; however, this strategy does not scale well to pairwise connections across the whole visual field, apparent separation depends on element size [10], and there is no clear account of why adaptation to dot textures should alter the interpretation of activity in the bi-local detector. In this demonstration, the test dot separation exceeds the mean dot separation of the adapting texture, so on a size or distance channels model [11], apparent separation would be predicted to increase.

**Figure 2:**
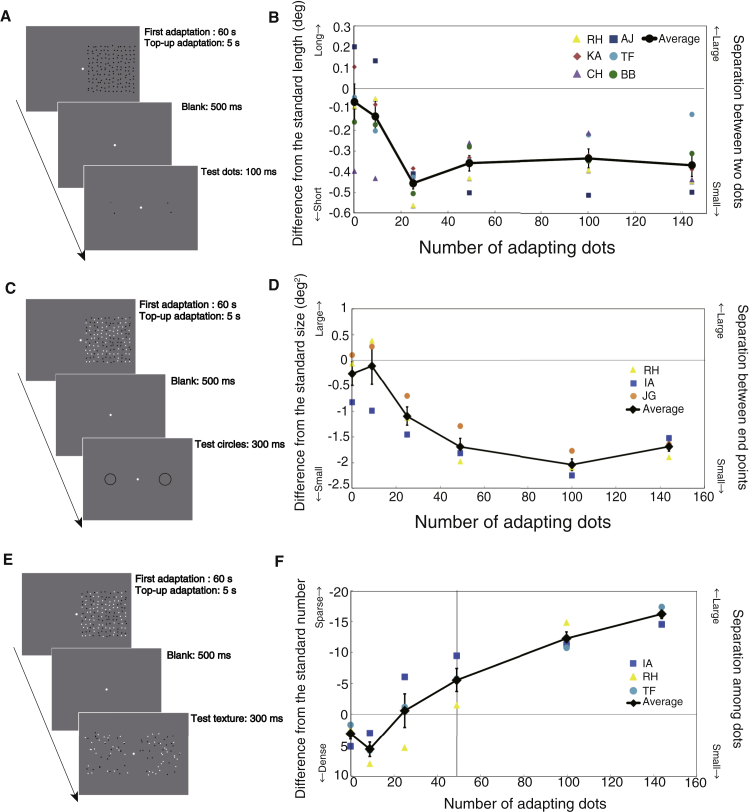
The Procedures for Stimulus Presentation and the Results in Experiments 1–3 (A–F) The procedures for stimulus presentation (A, C, and E) and the results (B, D, and F) in experiment 1 (A and B: dot separation judgment; see also Movie S1), experiment 2 (B and C: circle size judgment; see also Figure S1 and Movie S2), and experiment 3 (E and F: dot density judgment; see also Movie S3). The dashed lines indicate each participant’s data, and the bold line shows the average across participants. All error bars are ±1 SE. (A and C) In all trials, the comparison stimuli were presented in adapted area (cancelation method). (B and D) The vertical axis indicates the differences between the points of subjective equality and the standard length 4 deg (B) or the standard size 12.57 deg^2^ (D). (E) In all trials, the comparison stimuli were presented in non-adapted area (matching method). (F) The vertical axis indicates the difference between the point of subjective equality and the standard number of texture elements (49 dots). Positive values mean that the subject perceives a denser texture than 49 dots after adaptation. The rigid vertical line marks 49 adapting dots.

In order to establish the relationship between distance compression and density aftereffects in more detail, we systematically manipulated the number of adapting dots. The adapting texture, placed on one side of fixation, was a square array of black dots each of which had a random spatial displacement that was reset every 300 ms (Movie S1). The other side of fixation, in which the comparison dots were subsequently presented, was set to the background color. Perceived separation was measured using a standard binary choice psychophysical procedure (see Methods). The perceived distances between the two black test dots depended upon adapting dot number (Figure 2B). The apparent compression peaked at around 25 adapting dots, when the test separation matched the average dot density, after which the effect saturated. We also measured the change in apparent density for the same adaptor (Figure 1C; see also Movie S3). Figure 2F shows that adapting to a higher density than the test pattern makes the test texture appear sparser, while adapting to a lower density does not make the test texture appear more dense, or does so only weakly. Our result is in general consistent with the reports that the density aftereffect only introduces a reduction in perceived density [12, 13], unlike spatial frequency (SF) adaptation, which shows a clear repulsion effect for similar test SFs [11]. Comparison of Figures 2B and 2F clearly indicates that the same adaptor increases the texture dot sparseness, while compressing the dot separation, for a wide range of adaptor dot densities.

To test whether the apparent compression was specific to pairs of locations or more universal, we repeated the adaptation experiment with a circle (Movie S2). We measured the apparent size of the circle after adapting to textures that varied in density. Figure 2D shows that the perceptual shrinkage increased with the number of adapting dots, reaching a peak compression of around 15%. Adaptation induced a reduction in apparent size uniformly over the form (Figure 1E), suggesting a uniform rescaling within the adapted space rather than the mislocation of individually selected points. To guard against the possibility that the shifts in the point of subjective equality resulted from biased responses when participants were uncertain, we repeated the task with a third response option, which was the choice of “no difference between two stimuli” [14]. The shrinkage in apparent size was still clearly evident (see Figure S1), indicating a change in appearance rather than a change in response bias.

**Figure 3:**
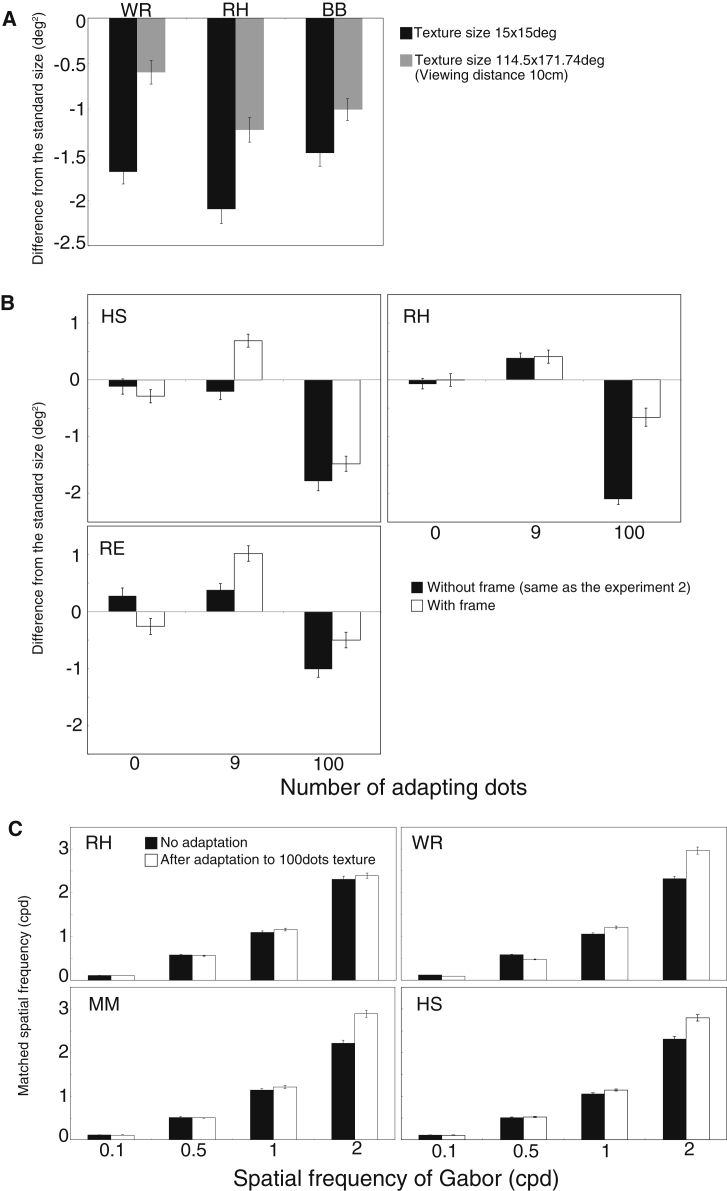
Results of the Control Experiments (A) A comparison of the degree of apparent shrinkage between the squared adapting texture at the 60-cm viewing distance and the large adapting texture (the vertical half of the monitor) with the 10-cm viewing distance. (B) A comparison of the degree of apparent shrinkage between with- and without-frame conditions. (C) The perceived spatial frequencies after density adaptation or no adaptation. In all figures, error bars are ±1 SE estimated from bootstrap method. See also Movie S4.

It is well known that apparent size can change after exposure to larger or small objects [15]. The window size for the adapting texture was generally larger than the circle on the adapted side. To remove any influence of the window, we constructed an adaptor that almost filled a visual hemifield, making made it difficult to see the edges of the texture. Figure 3A shows the effects of both small and large adapting textures on apparent circle size. Although the apparent shrinkage was greater for the windowed texture, indicating the window may contribute to the apparent size reduction in experiment 2, perceived compression remained when the adaptor covered the full hemifield, indicating a substantial random dot texture effect. To examine the window effect more closely, we conducted another experiment in which two black frames were presented with the adapting texture in both left and right visual fields during adaptation. As in experiment 2, observers judged circle size. Figure 3B shows the results for the explicit frame condition. If the window was the sole cause of the apparent shrinkage, the difference between the conditions should disappear, as an explicit window is present in all conditions. However, the perceived compression remained in the 100 dots condition, although the shrinkage was greater for the without-frame condition. For all subjects, even with the frames, the difference in shrinkage between the 9 and 100 dots conditions was clear. These results indicate that the perceived shrinkage after density adaptation is not due to the size aftereffect.

The density aftereffect does not appear to be easily explained on the basis of a shift in the activity of SF channels [12, 13]; however, density could in principle be computed, locally, as the ratio of the output of a high SF filter relative to that of a low SF filter [6, 7]. The high SF information provides a proxy for content, whereas the low SF information provides a proxy for area [7]. To check whether the effect of random dot texture adaptation on perceived size was linked to a reduction in apparent SF (more activity in the low-frequency range would lead to reduced density in this model), we replaced the circle in experiment 2 with a Gabor patch and measured perceived SF (Movie S4). Figure 3C shows that after adaptation, perceived SF in the low SF conditions was not changed. In some subjects, the apparent SF of a 2 cycles per degree (cpd) carrier appeared to be higher after adaptation to dense texture. However, more activity in the higher SF range should deliver an increase in perceived density rather than a decrease. In our observations, the window of the Gabor patch appeared to be smaller, whereas the apparent SF inside did not change. This experiment indicates that change in apparent density and size can occur without a concomitant change in apparent SF in low SF channels and that the representation of size (area) may lie at a stage beyond early visual filtering operations.

**Figure 4:**
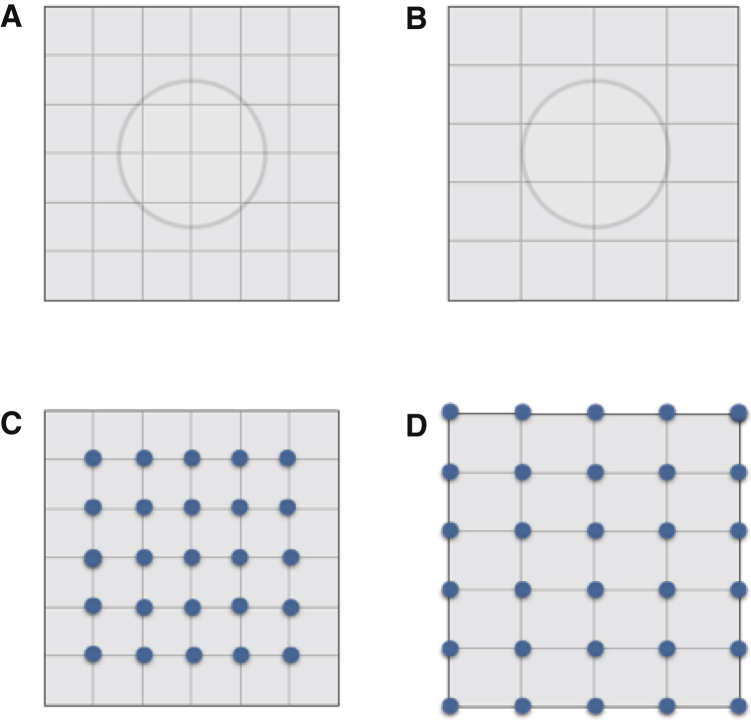
Illustration of the Visual Metric (A–D) After adaptation to random dot textures, there is an increase in a local neural measure that represents a unit area (A and C to B and D). Properties such as size are non-local and are referenced to this unit area, so this has the effect of the circle appearing to be smaller, i.e., have a diameter of two units rather than three (B). Texture density is a local visual property that mirrors the change in scale of the background against which the size and separation of foreground features such as geometric figures are judged (C and D).

Since size and separation can appear reduced after adaptation to high-density texture while at the same time textures appear sparser and apparent SF remains relatively unchanged, we cannot explain our observations on the basis of changes in sensitivity of a population of classical size or SF channels [11]. Separation is typically expressed relative to a metric (Figures 4A and 4C). The value one reports increases as the separation increases or as the unit of measurement decreases. Since the dots do not change location on the sensory surface, we propose that adaptation reduces the value of an explicit neural representation of local distance and area—an internal metric (Figures 4B and 4D). This representation can be thought of as a hypothetical neural signal that is referred to by control processes used to estimate distance or size. We envisage that this process would be akin to integrating neural signals expressing some modifiable elementary unit length along the path that separated the points to be compared, or integrating surface area elements in the case of size. We assume that adaptation to random dot texture leads to a subsequent reduction in the neural signal, as is the case for adaptation to contrast or speed [16]. The metric signal may also be modified in classic illusions such as the Oppel-Kundt [17, 18], but it does not appear to alter the scale of a dense texture, such as a sine grating. The random dot adaptation described above also reduces apparent density. Random dot texture density, unlike separation, but like color, motion, orientation, or SF, could be represented locally [6, 13]. We think of texture as revealing the metric properties of the fabric of visual space against which judgments of non-local geometric properties are made in the space-variant representations typical of biological vision systems.

## Experimental Procedures

### Methods

The stimuli were computer generated (Apple MacPro 2013) and displayed on a 22-in CRT monitor (1024 × 768 pixels, refresh rate 85 Hz, 2.24 min/pix, mean luminance 54.5 cd/m^2^, gamma corrected). The viewing distance was 60 cm, and the size of the adapting texture was 15° × 15° except in a control experiment in which the viewing distance was reduced to 10 cm. The duration of first adaptation period was 60 s with 5-s top-ups in subsequent trials. The adaptation and test periods were separated by a 500-ms gray field. The adapting texture dots (10 pixels diameter) were positioned relative to a square grid. Each dot was given random displacement of up to 30 arcmin, which was updated every 300 ms. The center of stimulus was 9.55° of visual angle from fixation. We used the binary choice method of constant stimuli to measure the point of subjective equality, with four sessions per data point. The adapted visual field was counterbalanced among sessions. There were breaks of at least 8 min between sessions. There were six adaptation conditions (0 [control], 9, 25, 49, 100, and 144 dots).

### Experiment 1

The adapting texture and test consisted of black dots. The duration of the test was 100 ms. The orientation of two test pairs was the same on a given trial but randomized across trials. The standard distance was 4°, and the comparison varied from 3° to 5° by steps of 0.33° (3° to 6° by steps of 0.5° for C.H.). The comparison was presented on the adapted side (cancelation method). The participant’s task was to report whether the left or right interval was longer.

### Experiment 2

The adapting texture consisted of equal numbers of white and black dots. Both the position and color of adapting dots were refreshed every 300 ms. The positions were spatially jittered as in experiment 1. The test was a black circle presented for 300 ms. The standard size was 12.57 deg^2^, and the comparison size was varied from 9.62 to 15.90 deg^2^ by steps of 0.94 deg^2^ for R.H. and J.G. and from 8.04 to 18.10 deg^2^ by steps of 1.40 deg^2^ for I.A. The comparison was presented on the adapted side (cancelation method). The participant’s task was to report whether the left or right circle was larger.

### Experiment 3

The adapting texture was the same as in experiment 2. The test was a texture that consisted of white and black dots with randomized positions. The test duration was 300 ms. The standard number of test dots was 49, and the comparison was varied from 10 dots to 88 dots in steps of 13 dots. The comparison was presented on the non-adapted side in this experiment (matching method). The participant’s task was to answer whether the left or right texture was denser.

### Experiment 4

Experiment 4 for was a replication of experiment 2 but with a large adaptor. The viewing distance was 10 cm, and the adapting stimulus subtended 114.5° × 171.74°. The visual angle of all test stimuli was the same as in experiment 2. Only the 100 dots adaptation condition was tested. The comparison was presented on the adapted side (cancelation method). The participant’s task was the same as in experiment 2.

### Experiment 5

Experiment 5 was a replication of experiment 2 but with an explicit frame present in both adapting fields. The procedure and stimuli were same as in experiment 2, but black square frames (16° × 16°, the width was 0.33°) were presented along with the adapting textures in both left and right visual fields to equalize the window effects. We used only 0, 9, and 100 dots conditions in this experiment.

### Experiment 6

The adapting texture was the same as in experiment 3. The test was a Gabor patch (the SD was 2°, and the Michelson contrast was 0.99). The standard SF was 0.1, 0.5, 1, or 2 cpd. The maximum SF of the comparison was two times the standard SF, and the minimum was ½ of the standard SF (in the 0.1 condition for W.R., the maximum was three times, and the minimum was 1/3). We used the 0 and 100 dots adaptation conditions. The comparison was presented on the non-adapted side (matching method). The participant’s task was to report whether the left or right texture had the higher SF.

## Author Contributions

Conceptualization, R.H. and A.J.; Methodology, R.H., S.N., and A.J.; Investigation, R.H.; Writing – Original Draft, R.H. and A.J.; Writing – Review & Editing, S.N. and A.J.

## Supplemental Information

Document S1. Supplemental Experimental Procedures and Figure S1

Movie S1. Demonstration of Experiment 1, Related to Figure 2 After observing the adapting texture, the interval between the two dots in the right visual field will appear shorter than the interval in the left visual field.

Movie S2. Demonstration of Experiment 2, Related to Figure 2 After observing the adapting texture, the size of the test circle in the left visual field will appear smaller than that in the right visual field.

Movie S3. Demonstration of Experiment 3, Related to Figure 2 After observing the adapting texture, the density of the text texture in the left visual field will appear to be sparser than that in the right visual field.

Movie S4. Demonstration of the Effect on Density Adaptation on Apparent Spatial Frequency, Related to Figure 3 After observing the adapting texture, the apparent spatial frequency of the test Gabors does not differ in the left and right visual fields.

Document S2. Article plus Supplemental Information
