# Supplementary material for: An Adaptable Metric Shapes Perceptual Space
Source: Curr Biol. 2016 Jul 25;26(14):1911–5. doi: 10.1016/j.cub.2016.05.047 (PMC4963211; doi:10.1016/j.cub.2016.05.047)
Supplement: Document S1. Supplemental Experimental Procedures and Figure S1 [file mmc1.pdf]

Current Biology, Volume *26*

Supplemental Information

An Adaptable Metric Shapes Perceptual Space

Rumi Hisakata, Shin'ya Nishida, and Alan Johnston

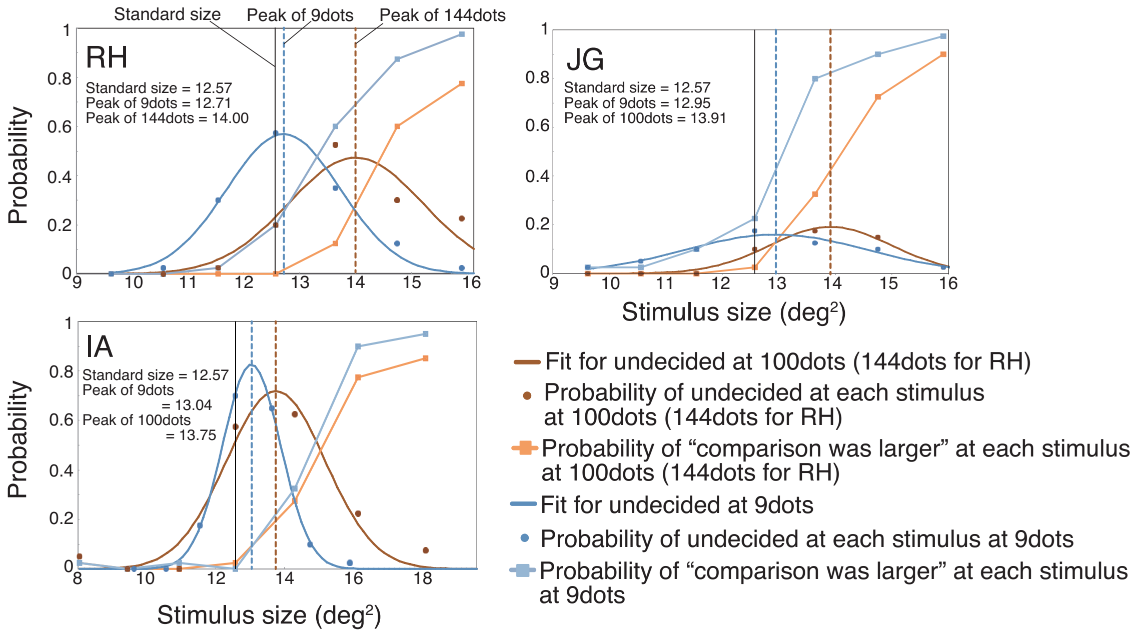

Figure S1: Result of the experiment with a third response option; related to Figure2. In the main experiments, the adapting texture was only presented in one half of the visual field. A tendency for participants to respond smaller on the adapted side when unsure could masquerade as a perceptual difference. To guard against this type of response bias we repeated the task in Experiment 2 with a third option, which was the choice of "no difference between two stimuli", following Garcia-Perez & Alcala-Quintana[S1]. The figure shows the response probabilities of answering "no difference" (blue/orange circles and functions) alongside psychometric functions derived from the larger and smaller decisions. The dots and the fitting curve indicate the probability of answering "no difference between the sizes of right and left circles". The peak no difference response probability in the dense adaptation condition shifted to a higher value relative the sparse adaptation condition. Thus a larger circle in the high density adaptor side appeared indistinguishable from a smaller circle on the low density adaptor side, indicating a change in appearance rather than a change in response bias.

Supplemental Experimental procedures

We repeated Experiment 2 with a third choice: subjects could report, "no difference between right and left". We used only the 9 and 100 dots (144 dots only for RH) adaptation conditions. A Gaussian function was fitted to the probability of answering "no difference" using the least-squares method. The comparison was presented on adapted side (cancelling method). The participant's task was to answer whether the left or right circle was larger, or whether there was no difference.

References

[S1] Garcia-Perez, M. A. & Alcala-Quintana, R. (2013). Shifts of the psychometric function: Distinguishing bias from perceptual effects. QJEP. *66,* 319-337.
