# Supplementary figures and images for: An Adaptable Metric Shapes Perceptual Space
Source: Curr Biol. 2016 Jul 25;26(14):1911–5. doi: 10.1016/j.cub.2016.05.047 (PMC4963211; doi:10.1016/j.cub.2016.05.047)

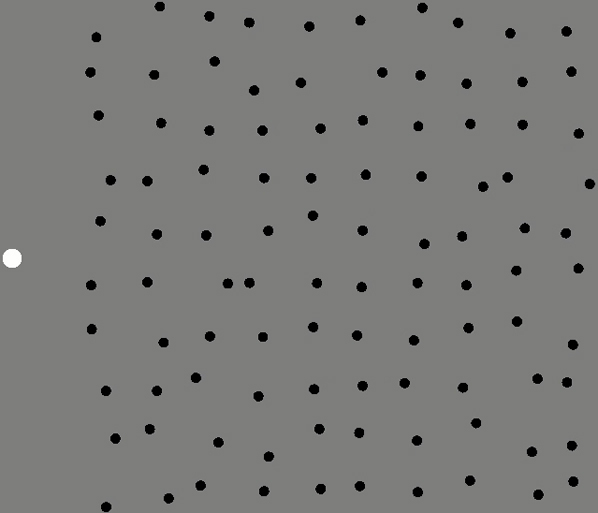

Supplement: Movie S1. Demonstration of Experiment 1, Related to Figure 2 — After observing the adapting texture, the interval between the two dots in the right visual field will appear shorter than the interval in the left visual field. [file mmc2.jpg]

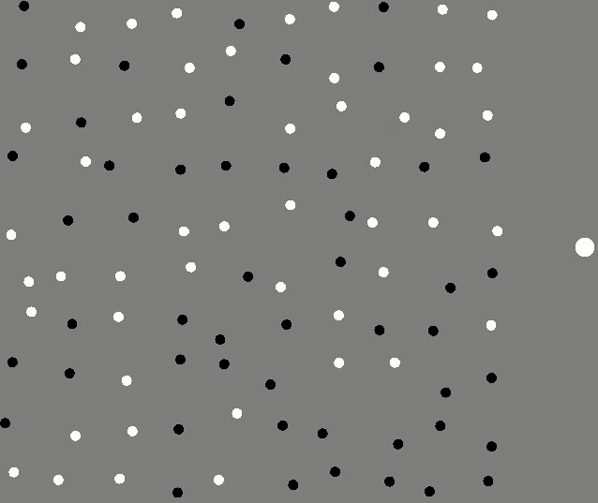

Supplement: Movie S2. Demonstration of Experiment 2, Related to Figure 2 — After observing the adapting texture, the size of the test circle in the left visual field will appear smaller than that in the right visual field. [file mmc3.jpg]

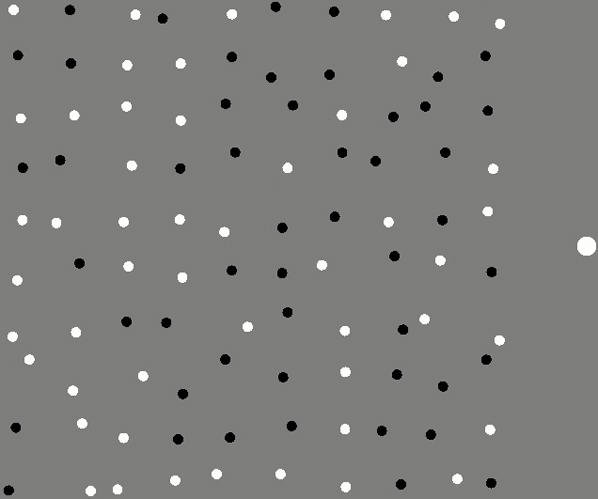

Supplement: Movie S3. Demonstration of Experiment 3, Related to Figure 2 — After observing the adapting texture, the density of the text texture in the left visual field will appear to be sparser than that in the right visual field. [file mmc4.jpg]

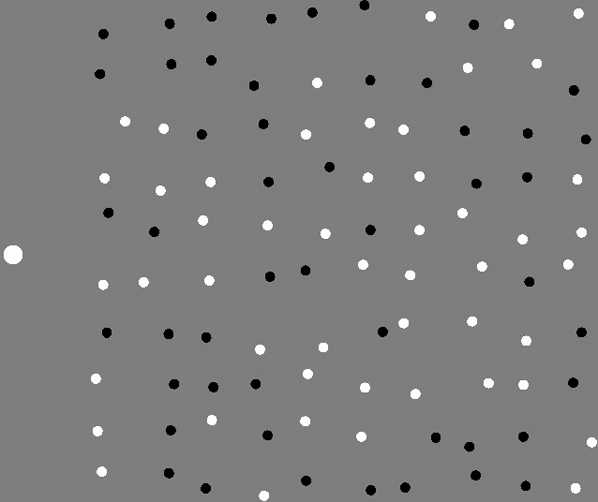

Supplement: Movie S4. Demonstration of the Effect on Density Adaptation on Apparent Spatial Frequency, Related to Figure 3 — After observing the adapting texture, the apparent spatial frequency of the test Gabors does not differ in the left and right visual fields. [file mmc5.jpg]
